# Acceptability, tolerability, and potential efficacy of cognitive behavioural therapy for Insomnia Disorder subtypes defined by polysomnography: A retrospective cohort study

**DOI:** 10.1038/s41598-018-25033-3

**Published:** 2018-04-27

**Authors:** Christopher B. Miller, Colin A. Espie, Delwyn J. Bartlett, Nathaniel S. Marshall, Christopher J. Gordon, Ronald R. Grunstein

**Affiliations:** 1 Big Health Ltd, London, UK; 2 CIRUS, Centre for Sleep and Chronobiology, Woolcock Institute of Medical Research, The University of Sydney, Sydney, NSW Australia; 3 Sydney Medical School, The University of Sydney, Sydney, NSW Australia; 4 Nuffield Department of Clinical Neurosciences and Sleep & Circadian Neuroscience Institute, University of Oxford, Oxford, UK; 5 Sydney Nursing School, The University of Sydney, Sydney, NSW Australia; 6 Department of Respiratory and Sleep Medicine, RPAH, Sydney Local Health District, Sydney, NSW Australia

## Abstract

In this retrospective cohort study, we describe acceptability, tolerability and potential efficacy of cognitive behavioural therapy (CBT) in Insomnia Disorder subtypes, derived from polysomnography (PSG): insomnia with normal-sleep duration (I-NSD) and insomnia with short-sleep duration (I-SSD). All research volunteers were offered access to digital CBT, single component sleep restriction therapy and face-to-face group CBT. Follow-up occurred at three months post-treatment using the insomnia severity index (ISI). 96 participants (61 females, mean age of 41 years) were grouped into either normal-sleep (n = 53) or short-sleep (n = 43). CBT was acceptable to 63% of participants (normal-sleep = 31, short-sleep = 29), with 28 completing therapy (tolerability: normal-sleep = 11, short-sleep = 17). For potential efficacy, 39 (normal-sleep = 20, short-sleep = 19) out of 96 participants (41%) completed a follow-up ISI assessment. In this reduced sample, mean (SD) ISI scores decreased across both groups (normal-sleep: 18.0 (4.0) to 10.7 (4.6); short-sleep: 16.5 (5.5) to 11.0 (6.3); both P < 0.01). Those with normal-sleep were more likely to respond (≥6-point ISI reduction) to CBT compared to short-sleep (70%, n = 14/20 vs. 37%, n = 7/19 respectively, P = 0.038). In this cohort, 60 (63%) of participants attempted CBT and of those 28 (47%) completed therapy. Results may be comparable to clinical participants with implications for the successful translation of CBT for insomnia.

## Introduction

Insomnia is a common and distressing disorder that impairs quality of life and affects approximately 6 to 10% of the adult population^1,2^. Research has focused on pharmacological (hypnotics) and non-pharmacological treatments with cognitive behavioural therapy (CBT) delivered either as face-to-face or internet-based therapy^1,3,4^. CBT is now considered as a first line intervention but lacks health system capacity for delivery to the insomnia population^5^. Stepped care models have been proposed as a potential solution promoting low-cost access to CBT^6–9^. Digital CBT would be the entry treatment modality where group and or one-on-one therapy with an expert Psychologist would be required when participants are non-responders^7^. Little assessment of CBT treatment modalities is available in the insomnia population especially when treatment is an addition to a research study, with different offered CBT modalities.

One aspect of who responds to CBT may be explained by differing insomnia subtypes. First-night polysomnography (PSG) has been used to identify an objective insomnia subtype with short-sleep duration (I-SSD: <6 hours total sleep time) that is biologically distinct from insomnia with normal-sleep duration (I-NSD: >6 hours total sleep time)^10^. Indeed, I-SSD has been linked to a range of medical and psychiatric co-morbidity, including adverse cardiometabolic outcomes, neurocognitive impairment and sleep-wake misperception^10^. Importantly, it has been proposed that this phenotype is clinically useful as participants may not respond as well to CBT treatment compared to participants with insomnia and more normal-sleep duration insomnia (>6 hours TST)^10^. I-SSD is therefore considered a more severe insomnia subtype which may be associated with a poorer treatment response to CBT^10^. Previously, Troxel and colleagues found baseline objective insomnia symptoms in depressed participants was a moderator of treatment (including pharmacotherapy and/or psychotherapy) remission^11^. A prolonged sleep latency and short sleep duration, independently or in conjunction with insomnia, was associated with risk of and non-remission of depression^11^.

Recently, two studies evaluated this hypothesis in secondary analyses of existing CBT randomized controlled trial data. Using a brief group-based CBT intervention in a group of older (ca. 65+ years) insomnia participants, no significant differences were found in treatment response between insomnia subtypes (CBT: n = 30 I-SSD; n = 33 I-NSD vs. waitlist: n = 9 I-SSD; n = 19 I-NSD - using ambulatory PSG and a cut-point of <6 hours)^12^. However other researchers found an attenuated treatment response in participants (n = 35 I-SSD; n = 25 I-NSD) with <6 hours sleep duration (actigraphy) for the insomnia symptom questionnaire^13^. Traditionally, ambulatory PSG and actigraphy have not been used frequently to define insomnia subtypes and may not be analogous to in laboratory-based PSG^14^. No study has as yet reported Insomnia Disorder (DSM-5) treatment response to CBT with laboratory-based PSG-determined I-SSD and I-NSD subtypes.

In this study, we aimed to provide initial pilot data describing the use of different CBT modalities to manage insomnia subtypes through a simple retrospective pre-to-post observational cohort study as part of feasibility testing. In this exploratory study we also aimed to describe CBT acceptability and tolerability by Insomnia Disorder subtypes. We hypothesized that I-SSD participants would have higher insomnia severity index (ISI) scores at follow-up with lower ISI response and remission rates compared to participants with I-NSD.

## Methods

### Research volunteers, cognitive behavioural therapy and treatment response

We report a retrospective cohort study of research volunteers with insomnia. Participants were initially recruited as part of a previous insomnia phenotyping project (Australia New Zealand Clinical Trials Registry (ANZCTR) number: ACTRN 12615000751572)^15^. At the end of this project, all participants were offered at least one CBT treatment modality as we were morally obligated to provide insomnia treatment to these participants. We then opportunistically followed-up participants to evaluate acceptability (number of participants who start a CBT treatment modality), tolerability (number of participants who completed a CBT treatment modality) and potential efficacy (response to treatment measured by the ISI) of therapy by objective insomnia subtype. In the initial research project, research volunteers with DSM-5 defined Insomnia Disorder^16^ were recruited through responses to clinic and local community advertising as described previously^15^. Participants underwent one overnight laboratory testing to determine sleep duration subtype and were classified into I-SSD or I-NSD groups from PSG, in line with previous I-SSD literature^14,15^. All participants were initially offered free access to standardized 12-week digital CBT consisting of six sessions: Sleepio.com^TM ^^17^. Participants were also informed that they could opt for face-to-face CBT with a Sleep Psychologist if they wished at reduced cost by obtaining a Mental Health Treatment Plan from their Family Physician (Better Access, Australia, 2006). For these participants, face-to-face group CBT (5 sessions) was delivered by at least a postgraduate level psychologist. A sub-sample of participants recruited a quarter to midway of the total recruited (participants numbers 24-66 out of 100) were offered single component sleep restriction therapy (SRT) over five weeks as part of another study without any other treatment^18^. At the end of SRT, participants were then also offered access to digital or group CBT.

We assessed the number of participants who started any CBT intervention by insomnia subtype. Participants were coded as either receiving CBT or treatment unknown (did not access digital CBT, face-to-face CBT at our clinic or SRT). We then explored proportions of insomnia subtypes who started either individual therapy (face-to-face CBT, digital CBT or SRT) or those who pragmatically went on to start a combined form of therapy (face-to-face CBT + digital CBT, face-to-face CBT + SRT or digital CBT + SRT). For tolerability, we assessed proportions of CBT completers (defined as 5 face-to-face CBT sessions, 6 sessions of digital CBT or 5 weeks of SRT) by insomnia subtype for those who started any form of CBT only. Potential efficacy was assessed using follow-up ISI scores only for this report (primary outcome). Participants were asked to complete an electronic version of the ISI^19^ at baseline and 3 months post-CBT. The ISI has been validated as a clinical insomnia treatment outcome and can classify insomnia participants as responding to treatment (≥6-point reduction from baseline) and remitting from insomnia (<8 points)^20,21^. All volunteers gave written informed consent and ethics for the initial phenotyping research and follow-up assessment was approved by the Royal Prince Alfred Hospital Ethics Review Committee, Sydney, Australia (Protocol No X11-0392 & HREC/11/RPAH/620). All methods were carried out in accordance with the approved guidelines.

### Statistical analysis

Cluster analysis was used to classify participants as either I-SSD or I-NSD. Subtypes were built from first-night PSG-derived sleep parameters including total sleep time, sleep onset latency, and wake-time after sleep onset^15^. Cluster analysis provides non-subjective demarcation of participant cut-points from PSG^22^. We report an intention-to-treat type analysis (where all those with PSG data were analysed for follow-up outcomes) and not a per-protocol type analysis intention-to-treat is more reflective of what may happen to insomnia participants in clinical settings which we were keen to understand. Paired sample t-tests evaluated overall and insomnia subtype treatment response using the ISI and boxplots and histograms assessed normality. χ^2^ tests evaluated insomnia subtype treatment response and remission rates from the ISI. For a *d* = 0.5 treatment effect size, we were initially powered (G*Power 3.1) at 78% from the 96 participants (I-NSD n = 53 and I-SSD n = 43) at baseline. However, only 39 participants (I-NSD n = 20 and I-SSD n = 19) completed the follow-up ISI, reducing power to 43%. Therefore, it was not possible to confidently compare subtypes statistically using analysis of variance because of low numbers of follow-up data. All data were analyzed (by CBM) using SPSS software (IBM v 24.0.0; IBM Corp, Armonk, NY, USA).

### Data availability statement

All data generated or analysed during this study are included in this published article (and its Supplementary Information files).

## Results

Demographic, sleep parameters (used to cluster insomnia participants)^15^, clinical information for each insomnia subtype I-NSD (n = 53) and I-SSD (n = 43) are presented for the 96 participants with Insomnia Disorder in Table 1.

**Table 1:** Means (standard deviations) and N (proportion) for demographic, sleep parameters (used to cluster individuals) and clinical features of the sample.

	Overall (n = 96)	I-NSD (n = 53)	I-SSD (n = 43)	P
Age (y), mean (SD),	41.4 (11.8),	38.6 (11.6),	44.8 (11.2),	0.010
range	23–75	23–66	23–75	
Sex (f)	61 (64%)	36 (68%)	25 (58%)	0.322
Insomnia severity index	17.3 (4.8)	18.0 (4.0)	16.5 (5.5)	0.154
Total sleep time (mins)	346.1 (67.8)	392.3 (35.4)	289.2 (53.4)	.
Sleep onset latency (mins)	25.3 (23.9)	18.0 (11.7)	34.32 (31.2)	.
Wake-time after sleep onset (mins)	72.5 (58.6)	39.9 (22.6)	112.6 (64.4)	.

Note: Total sleep time, sleep onset latency and wake-time after sleep onset are different by design of the cluster groups and are not tested for statistical significance. CBT: cognitive behaviour therapy; I-NSD: Insomnia with normal sleep duration; I-SSD: Insomnia with short sleep duration.

### Acceptability and Tolerability

**Figure 1:**
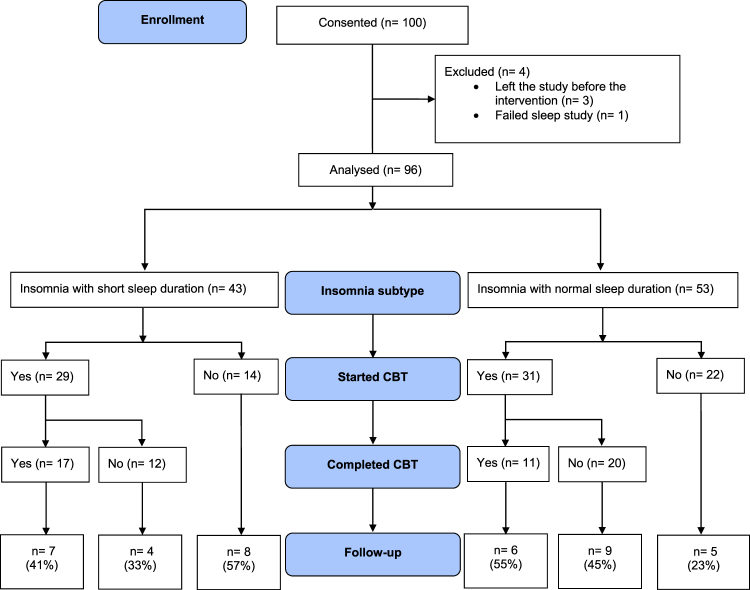
Study flow diagram with results of participants by insomnia subtype through psychotherapy treatment for Insomnia Disorder. The follow-up section displays the number (%) of participants who completed this assessment by insomnia subtype. CBT: Cognitive behavioural therapy; I-NSD: Insomnia with normal sleep duration; I-SSD: Insomnia with short sleep duration.

For acceptability, 60 participants (63%) from 96 overall, started therapy with 31/53 (59%) from I-NSD and 29/43 (67%) from I-SSD: see Fig. 1. For tolerability, 28 completed therapy with 11/53 (21%) from I-NSD and 17/43 (40%) from I-SSD. Participant completion rates and proportions of participant selected CBT treatment modality are presented in Table 2.

**Table 2:** Completion rates and uptake for participant selected cognitive behavioural therapy treatment modality.

	Completion rate (%) proportion (n) [95% CI]
Digital CBT only	47% (7/15) [21,73]
Face-to-face CBT only	26% (7/27) [11,46]
Digital CBT + face-to-face CBT	71% (5/7) [29,96]
SRT only	100% (6/6) [54,100]
SRT + face-to-face CBT	75% (3/4)
SRT + digital CBT	0% (0/1)

Note: From 96 participants, 60 started at least one form of therapy for insomnia, 28 completed therapy (defined as 5 face-to-face CBT sessions, 6 sessions of digital CBT or 5 weeks of SRT), and 36 did not start therapy at our clinic. Completion rates are expressed as a percentage of the number of participants who completed against those who started CBT by treatment modality with 95% confidence intervals. Confidence intervals and are not calculated when the numerator is 3 or fewer because the rate is not statistically distinguishable from zero. CBT: cognitive behaviour therapy; I-NSD: Insomnia with normal sleep duration; I-SSD: Insomnia with short sleep duration; SRT: sleep restriction therapy.

### Potential efficacy

In the thirty-nine (I-NSD = 20/53 (38%), I-SSD = 19/43 (44%)) out of 96 (41%) participants who completed the post-treatment ISI assessment (see Table 3), CBT was effective as mean ISI (SD) scores reduced from 17.4 (5.7) to 10.8 (5.4), (P < 0.001). For subtypes, I-NSD scores reduced significantly from 18.0 (4.0) to 10.7 (5.5), (P < 0.001), and I-SSD reduced from 16.5 (5.5) to 11.0 (6.3), (P < 0.01).

**Table 3:** Baseline-three months post-cognitive behavioural therapy follow-up insomnia severity index scores for each insomnia subtype group.

	Baseline					Three months post-CBT follow up				
	n	$\bar{{\rm{x}}}$	^95%^CI$\bar{{\rm{x}}}$	SD	^95%^CI_SD_	n	$\bar{{\rm{x}}}$	^95%^CI$\bar{{\rm{x}}}$	SD	^95%^CI_SD_
I-NSD	53	18.0	16.9, 19.1	4.0	3.3, 4.9	20	10.7	8.6, 12.9	4.6	3.5, 6.7
I-SSD	43	16.5	14.8, 18.2	5.5	4.6, 7.1	19	11.0	7.9, 14.0	6.3	4.8, 9.3

Note: CBT: Cognitive behavioural therapy; CI: Confidence interval; I-NSD: Insomnia with normal sleep duration; I-SSD: Insomnia with short sleep duration; SD: Standard deviation.

### Insomnia Response and Remission

**Figure 2:**
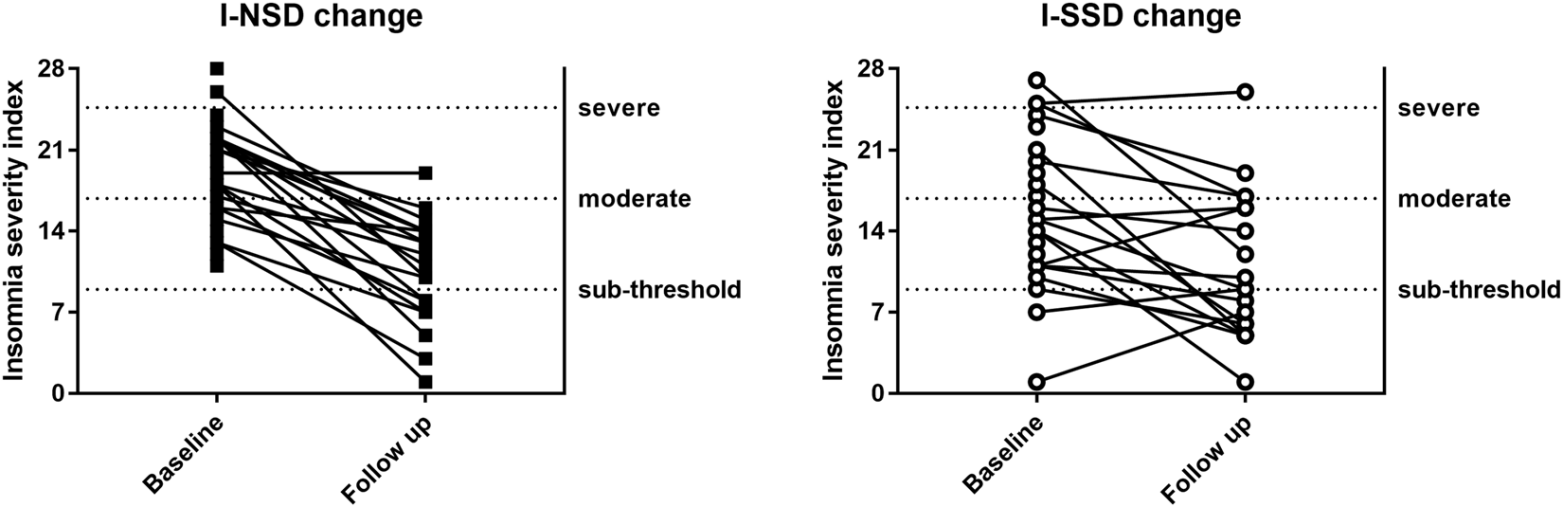
Insomnia severity index scores for all participants by subtype who underwent overnight polysomnography linked before and after three months cognitive behavioural therapy. I-NSD: Insomnia with normal sleep duration; I-SSD: Insomnia with short sleep duration.

Participants with I-NSD were more likely to respond (≥6 ISI point reduction from baseline) to CBT: 70% (n = 14/20, 95% CI [46, 88]) compared to I-SSD 37% (n = 7/19, 95% CI [16, 62]), (χ^2^ = 4.31, P = 0.038). There were no differences for insomnia remission (<8 ISI points at follow up) rates (I-NSD = 30% (n = 6/20, 95% CI [12, 54]), I-SSD = 32% (n = 6/19, 95% CI [13, 57]), (χ^2^ = 0.01, P = 0.915). Figure 2 shows charts of all individual participant ISI observations before and after three months post CBT for each subtype.

## Discussion

This retrospective cohort study aimed to describe the acceptability, tolerability and potential efficacy of CBT treatment modalities (digital and face-to-face) in participants with normal sleep duration (I-NSD) and short sleep duration insomnia (I-SSD) defined using in-laboratory polysomnography (PSG)^14,15^. Initial observational data suggests CBT uptake was acceptable for most participants (63%) across both subtypes with significant improvements in ISI scores. Previous research found participants with short sleep insomnia were less likely to respond to CBT, whilst those with I-NSD had a better treatment response^13^. Interestingly, our findings as per Fig. 2 appear to reflect a similar trend with a more consolidated response to treatment in the I-NSD participants compared with considerably more variability in the treatment response to CBT seen in the I-SSD. Insomnia remission rates were not different between subtypes. However, due to a lack of follow-up data, we were unable to test for mean differences at post-treatment. Treatment-related results evaluating potential efficacy should therefore be interpreted with caution due to missing data. Choice of CBT treatment modality may explain why both insomnia groups had similar remission rates and requires further investigation in a randomised controlled treatment study.

This was an initial study and longitudinal follow-up over a longer time period would provide insight whether I-SSD is more difficult to treat compared with I-NSD participants. Only 41% of participants (I-SSD: n = 19; I-NSD: n = 20) completed the follow-up ISI assessment. This may reflect what may be observed more clinically with insomnia management where participants were offered therapy options as compared with a more standardised randomised controlled trial. Different delivery modes of CBT consisted of varying treatment lengths and may also have affected results. Therefore, this study is exploratory requiring more power in future clinical trials. We calculated that we were powered at 78% to detect a between-groups treatment effect size of *d* = 0.5 which was reduced to 43% because of missing follow-up data. To aid future studies, we report the 95% confidence limits of both the means and standard deviations in Table 3. These suggest that differences between insomnia subtypes are probably small to detect (*d* ≥ 0.3) for the insomnia severity index. Therefore, we estimate that for future clinical trials with 80% power around 182 participants are required in total (based on a 1:1 allocation ratio).

All participants were unpaid but given free access to digital CBT choosing whether-or-not to use this and/or to receive face-to-face CBT at our sleep centre. Treatment uptake was based solely on participant preference with participants interacting with the intervention delivery type that may have suited them best. We believe this reflects a more clinically valid situation with participant choice which may occur under a stepped care type framework. What is concerning is that 36 participants from a total of 96 recruited did not commence any form of CBT. This may have been due to a number of factors including readiness to undertake therapy, access to face-to-face therapy through a Mental Health Care Treatment Plan, and lack of clinical support in an automated digital CBT. Further to this, only 28 participants out of 96 completed therapy which requires feasibility assessment and this figure was different across treatment modalities (e.g., SRT). It may be that certain therapies are more acceptable to different participants or subtypes and further research should evaluate this. Our defined treatment-related constructs of acceptability and tolerability were inferred outcomes. From these data, it was not possible to understand specific reasons for participant treatment attrition (e.g., lack of treatment acceptability, tolerance or efficacy). The ISI was the primary endpoint here and further prospective studies should look to measure PSG-defined sleep duration as the primary outcome. Pre-defined clinical trials should now evaluate differences in both subjective and objective outcomes between insomnia subtypes in response to treatment. Gathering this information would no doubt provide insight into insomnia phenotype-specific interventions, where a combination of treatments may be more valid possibly using short-term pharmacotherapy^10,14^. We did not examine individual CBT which is considered the most commonly used psychotherapy for insomnia.

## Conclusion

Cognitive behavioural therapy delivered in a digital or face-to-face format is acceptable to most participants (63%) with Insomnia Disorder. However, of participants who started CBT, only 47% completed therapy. For those 39 participants who completed the follow-up assessment, CBT appeared efficacious for subjective insomnia severity. Participants with insomnia and normal sleep duration appear to respond better compared to those with insomnia and short sleep duration.

## Electronic supplementary material

Dataset
